# Immune Dysfunction and Autoimmunity as Pathological Mechanisms in Autism Spectrum Disorders

**DOI:** 10.3389/fncel.2018.00405

**Published:** 2018-11-13

**Authors:** Heather K. Hughes, Emily Mills Ko, Destanie Rose, Paul Ashwood

**Affiliations:** ^1^Department of Medical Microbiology and Immunology, University of California, Davis, Davis, CA, United States; ^2^MIND Institute, UC Davis Medical Center, Sacramento, CA, United States

**Keywords:** autism, immune, dysregulation, autoimmunity, neurodevelopment, behavior

## Abstract

Autism spectrum disorders (ASD) are a group of heterogeneous neurological disorders that are highly variable and are clinically characterized by deficits in social interactions, communication, and stereotypical behaviors. Prevalence has risen from 1 in 10,000 in 1972 to 1 in 59 children in the United States in 2014. This rise in prevalence could be due in part to better diagnoses and awareness, however, these together cannot solely account for such a significant rise. While causative connections have not been proven in the majority of cases, many current studies focus on the combined effects of genetics and environment. Strikingly, a distinct picture of immune dysfunction has emerged and been supported by many independent studies over the past decade. Many players in the immune-ASD puzzle may be mechanistically contributing to pathogenesis of these disorders, including skewed cytokine responses, differences in total numbers and frequencies of immune cells and their subsets, neuroinflammation, and adaptive and innate immune dysfunction, as well as altered levels of immunoglobulin and the presence of autoantibodies which have been found in a substantial number of individuals with ASD. This review summarizes the latest research linking ASD, autoimmunity and immune dysfunction, and discusses evidence of a potential autoimmune component of ASD.

## Introduction

First defined as a distinct syndrome in 1943 by child psychiatrist Kanner (1943), autism spectrum disorders (ASD) are a group of heterogeneous neurological disorders that are clinically characterized by deficits in social interactions, communication, and stereotypical behaviors (Baio et al., 2018). Recent prevalence of ASD has risen dramatically to 1-in-59 US children, with preponderance toward males (Baio et al., 2018). Although increased awareness of the disorder and changing diagnostic criteria have undoubtedly contributed to the increase in prevalence (King and Bearman, 2009), researchers agree that these cannot solely account for such a significant rise in occurrence (Hertz-Picciotto and Delwiche, 2009). While the cause of the majority of ASD remains elusive, it likely involves a combination of genetic, epigenetic, and environmental factors. Twin studies have found a high concordance rate among monozygotic twins, with a much lower rate among dizygotic twins, implicating genetics as a factor; however, when combined, genetic interactions/mechanisms account for only 10–20% of ASD cases (Abrahams and Geschwind, 2008), this may suggest other heritable factors or shared genetic and environmental influences may be involved. These genetic markers may also be present in healthy individuals, again suggesting other risk factors in the pathogenesis of most cases of ASD. The rate for dizygotic twins is higher than non-twin siblings, and may suggest that shared prenatal environmental factors such as maternal infection, diet, and household chemical exposures may play a significant role in the etiology of ASD (Hallmayer et al., 2011). Environmental factors during pregnancy including maternal inflammation, air pollution and pesticide exposure have been associated with an increased risk of developing ASD and could be responsible for epigenetic changes identified in ASD (Loke et al., 2015); however, no single etiological factor has risen to the forefront. Research in ASD is confounded by the fact that these are highly heterogeneous disorders that likely have multiple etiologies and converging pathophysiological pathways. There is a growing need to determine which factors might be involved in the development of these disorders, as individuals with ASD and their families experience increased stress and decreased quality of life (Estes et al., 2013; Kuhlthau et al., 2014), and the increased prevalence of ASD has created a significant burden on health care (Lavelle et al., 2014) with an economic impact projected to exceed $400 billion by 2025 (Leigh and Du, 2015).

Although the specific etiologies of ASD remain unknown, many hypotheses regarding causation of ASD abound, including the potential involvement of the immune system. Just over a decade ago, we hypothesized that ASD may in fact be an autoimmune disorder (Ashwood and Van de Water, 2004; Ashwood et al., 2006). At that time, immune studies were limited and results had been somewhat inconsistent between various laboratories. Since then, a significant amount of research linking ASD and aberrant immune function has taken place, and although inconsistencies still exist, a clearer picture of the importance of immune involvement in ASD has emerged. This comprehensive review summarizes the latest research linking ASD and immune dysfunction, and discusses evidence of potential autoimmune mechanisms of ASD.

## Familial autoimmunity

Diverse autoimmune diseases within a nuclear family suggest familial autoimmunity (Anaya et al., 2007). Historically, studies have shown an increased prevalence of familial autoimmune disorders in ASD. The first study to identify a connection was in 1971, where researchers found a child with ASD who had several family members with multiple autoimmune disorders (Money et al., 1971). A later study found that 46% of ASD families had two or more members with autoimmune disorders, and as the number of autoimmune disorders within the family rose from one to three, the odds ratio for a risk of a child with ASD increased from 1.9 to 5.5. This study found that autoimmune prevalence was significantly increased in mothers and first-degree family members of ASD subjects, with type I diabetes (T1DM), rheumatoid arthritis (RA), hypothyroid and systemic lupus erythematosis (SLE) being the most common disorders found (Comi et al., 1999). A 2003 study also found a specific link to hypothyroid/Hashimoto's thyroiditis and RA in parents with ASD offspring (Sweeten et al., 2003a); however, others only found such an increase was paternally linked (Micali et al., 2004).

Since then, both self-reported and registry-based studies have shown additional links to autoimmunity in the family of children with ASD. A study in 2006 found autoimmune thyroid disease to be a significant risk factor, especially if the family member was the mother (Molloy et al., 2006a). This and other self-reported studies have some limitations as they may include biased or inaccurate recall. Utilization of health registries have allowed researchers to obtain larger datasets from patients based on medical records in population based case-control studies. In 2007, Mouridsen et al. found maternal ulcerative colitis and paternal T1DM were associated with infantile autism, (Mouridsen et al., 2007). In addition, mothers with autoimmunity were more likely to have a child with intellectual disability (ID), defined as an IQ of less than 50 (Mouridsen et al., 2007). In a large nationwide study that included all children born in Denmark from 1993 to 2004 (689,196 children, 3325 with ASD), researchers found that maternal RA and celiac disease increased risk of ASD in offspring (RA incidence rate ratio (IRR): 1.70 [95% CI: 1.07–2.54]; celiac disease IRR: 2.97 [95% CI: 1.27–5.75]), and an increased risk of infantile autism was found to be associated with family history of T1DM in either parent [IRR:1.78; 95% CI:1.16–2.61] (Atladottir et al., 2009). A further study utilizing the Danish registries looked specifically at both maternal and paternal RA and risk for ASD through 2007. Their data show that the risk of ASD increased by approximately 30% in cases of parental RA (maternal: HR 1.31 and 95% CI 1.06–1.63; paternal: HR 1.33, 95% CI 0.97–1.82). They conclude that a genetic component, specifically the Human Leukocyte Antigen − antigen D Related (HLA−DR) B1^*^04 alleles found commonly in both RA and ASD, may be playing a significant role in the development of ASD along with environmental factors (Rom et al., 2018). Registry-based studies have also shown an almost 50% higher odds-ratio of a child having an ASD diagnosis by age 10 if either parent had any autoimmune disease (maternal OR = 1.6 [95% CI = 1.1–2.2]; paternal OR = 1.4 [95% CI = 1.0–2.0]), with maternal T1DM, idiopathic thrombocytopenic purpura (ITP), myasthenia gravis and rheumatic fever carrying the highest risks (Keil et al., 2010).

Confirming the link to autoimmune thyroid disorders previously found in case reports, a nested case control study in Finland identified a significantly increased risk of developing ASD in children who were born to mothers positive for anti-thyroid peroxidase antibodies (TPO-Ab+) during pregnancy. This study looked at a population of children born in Finland between 1987 and 2005, which included 1,132 confirmed cases of childhood autism. When compared to 967 matched controls, they found odds for developing ASD without ID were increased nearly 80% among children born to mothers who were TPO-Ab+ during pregnancy (OR = 1.78, 95% CI = 1.16–2.75). Interestingly, the odds were not increased for development of ASD with comorbid ID (Brown et al., 2015).

These large population studies rely on medical reporting and abstraction of medical data, as well as only reporting past and present autoimmunity, therefore they may not cover younger mothers who develop autoimmunity after the study period has ended. Nonetheless, a consistent pattern has emerged, as multiple meta-analyses have confirmed the familial autoimmunity and ASD link. A 2016 meta-analysis of mainly cased-control studies found significant positive associations of ASD with maternal autoimmunity during pregnancy (pooled OR: 1.34, 95% CI 1.23–1.56) and maternal autoimmune thyroid conditions (pooled OR: 1.29, 95% CI 1.14–1.45) (Chen et al., 2016). Familial T1DM and autoimmune thyroid disease were also associated with higher rates of regressive autism, versus those with developmental delays evident during infancy (Scott et al., 2017). Overall, combined family history of autoimmune disorders increased the risk of ASD by 28%, with most significant increased risks associated with psoriasis 59%, RA 51%, T1DM 49%, and hypothyroid 64% (Wu et al., 2015)].

In summary, the results of these familial studies do not single out one autoimmune disease and risk for ASD; however, a clear familial autoimmune component has emerged. The significant overlap of autoimmunity within the family members of ASD subjects may suggest an involvement of inherited immune factors. Maternal autoimmunity could also be playing a role in the gestational immune environment that has been found to significantly influence neurodevelopment. Summary of these studies can be found in Table 1.

**Table 1:** Studies identifying association of familial autoimmunity and ASD.

**Subject details**	**Methods**	**Summarized findings**	**References**
61 ASD 46 TD	Self-reported questionnaire: known AI within family	46% ASD families had 2+ members with AI ↑ ASD odds ratio (1.9 to 5.5) as number of family members with AI ↑ from 1-3 ↑ T1DM, RA, hypothyroid and SLE in ASD mothers and 1st degree relatives	Comi et al., 1999
101 ASD 101 AI 101 HC	Self-reported questionnaire: which 1st and 2nd degree relatives have AI	↑ frequency of AI in ASD families compared to AI and TD families ↑ AI including hypothyroid/Hashimoto's thyroiditis and RA in ASD parents	Sweeten et al., 2003a
79 ASD 61 DD	Self-reported questionnaire: ASD with familial AI and psychiatric history	No significant relationship of AI in ASD vs. DD31.5% of ASD fathers vs 18.2% control had AINo difference found in mothers.	Micali et al., 2004
153 ASD 155 regressive ASD	Telephone interview: AI in 1st and 2nd degree relatives.	ASD with regression ↑ in families with 1st or 2nd degree AI relatives ↑ risk with familial AI thyroid diagnoses, especially maternal family members	Molloy et al., 2006a
111 ASD 330 TD	Registry based: Danish national hospital registry	↑ risk of ASD with maternal UC and paternal T1DMMothers with AI more likely to have child with ID (IQ < 50)	Mouridsen et al., 2007
3325 ASD (1089 “infantile autism”)	Registry based: all children born in Denmark 1993–2004	↑ risk of ASD with maternal RA and celiac disease ↑ risk of infantile autism associated with family history of T1DM, both parents.	Atladottir et al., 2009
1227 ASD 30,675 TD	Registry based: Three Swedish registries	↑ risk of ASD with AI in both parents ↑ risk of ASD with maternal T1DM, ITP, myasthenia gravis and rheumatic fever	Keil et al., 2010
967 ASD 967 TD	Nested case–control design: prospectively drawn maternal sera samples with registry-based ASD diagnoses from FiPS-A	↑ risk of ASD with maternal TPO-Ab presence during pregnancy	Brown et al., 2015
11 studies	Systematic review and meta-analysis	↑ risk of ASD with family history of all AI ↑ risk of ASD with familial hypothyroidism, RA, and psoriasis	Wu et al., 2015
10 studies	Systematic review and meta-analysis	↑ risk of ASD with maternal AI developed during pregnancy ↑ risk of ASD with maternal thyroid disease	Chen et al., 2016
206 ASD 33 regressive ASD	Medical chart review of ASD diagnosis and familial AI association	↑ risk of regressive ASD with familial AI Regressive ASD associated with familial T1DM and autoimmune thyroiditis	Scott et al., 2017

*ASD, autism spectrum disorders; TD, typically developing; AI, autoimmunity; T1DM, Type 1 diabetes mellitus; RA, rheumatoid arthritis; C: SLE, systemic lupus erythematosus; healthy control; BAP, broad autism phenotype; IBD, inflammatory bowel disease; UC, ulcerative colitis; ID, intellectual disability; ITP, idiopathic thrombocytopenic purpura; FiPS-A, Finnish Prenatal Study of Autism; TPO-Ab, thyroid peroxidase antibody*.

## Gestational immune influences

### Maternal autoantibodies

The gestational environment is protected by the placenta, a selective barrier that allows for nutrient uptake and waste elimination, and provides protection from pathogens while allowing protective immune factors such as immunoglobulin-G (IgG) to cross into the amniotic fluid compartment (Garty et al., 1994). This passage could be facilitating the transfer of maternal IgG that target fetal brain antigens and could play an etiological role in ASD by blocking or activating proteins in the fetal brain, or initiating a cascade of neuroinflammation. In addition to an increased prevalence of familial and maternal autoimmunity in ASD, a subset of mothers of children with ASD (10–12%) have been found to harbor autoantibodies with reactivity to fetal brain components (summarized in Table 2), and these antibodies induce ASD-like pathology in animal models (Martin et al., 2008; Braunschweig et al., 2012b; Bauman et al., 2013).

**Table 2:** Studies identifying presence of anti-brain autoantibodies in mothers of children with ASD.

**Subject details**	**Methods**	**Summarized findings**	**References**
Mother with 2 ASD children	IHC of sera binding to rodent brain.	Pilot study-maternal sera had reactivity to rodent Purkinje cells in cerebellum and large brain stem neurons.	Dalton et al., 2003
11 ASD mothers 10 TD mothers	Serum reactivity to prenatal, postnatal, and adult rat brain proteins by immunoblotting	↑ reactivity to prenatal rat brain in multiple patterns of low kDa weight, and one significantly higher at 250 kDa No reactivity to postnatal or adult rat brain	Zimmerman et al., 2007
100 ASD mothers 100 TD mothers	Serum reactivity to human and rodent fetal and adult brain tissues, GFAP, and MBP by immunoblotting	↑ reactivity at 36 kDa in both human fetal and rodent embryonic brain tissue. ↑ reactivity at 61 kD in human fetal brain tissue. ↑ reactivity at 36 and 39 kDa against human fetal brain in mothers whose children had regressive ASD.	Singer et al., 2008
61 ASD mothers 62 TD mothers 40 DD mothers	Plasma reactivity to human fetal and adult brain proteins by immunoblotting	↑reactivity to 73kDa and 37kDa to human fetal brain correlated with regressive ASD Reactivity to 37 kDa was higher in ASD mothers compared with TD and DD mothers. No reactivity with TD plasma to either tissue type.	Braunschweig et al., 2008
84 ASD mothers 49 DD mothers 160 TD mothers	Mid-pregnancy plasma reactivity to fetal brain protein by immunoblotting	↑reactivity at 39 kDa in ASD compared to DD and TDReactivity at both 39 kDa and 73 kDa seen only in early-onset ASD	Croen et al., 2008
202 ASD mothers 163 TD mothers	PCR for MET rs1858830 allele genotyping. Measured MET protein and cytokines by Luminex from stimulated maternal PBMCs. Previous study results used for the associations to presence of auto-Abs	Presence of C allele associated with reactivity at 37 and 73-kDa to fetal brain proteins. Presence of C allele associated with ↓ MET protein expression and ↓ IL-10	Heuer et al., 2011
277 ASD (70 BAP) 189 age-matched TD (2-5 years) and their mothers	Maternal plasma reactivity to Rhesus macaque fetal brain protein medleys by immunoblotting, child plasma reactivity to Rhesus macaque cerebellum protein medley.	↑ reactivity to many including proteins with MW of 42, 49, 60, 80, and 100 kDa in plasma from ASD mothersNo correlation with reactivity found in childrenChild results listed in Table 4.	Goines P. et al., 2011
204 ASD mothers 71 BAP mothers 102 DD mothers 183 TD mothers	Maternal plasma reactivity to Rhesus macaque brain at 3 gestational ages by immunoblotting	↑ paired reactivity at 37 and 73 kDa combined in ASD, not seen in TD ↑ paired reactivity at 39 and 73 kDa in ASD and BAP compared to TD and DD Paired reactivity at 37 and 73 kDa associated with language deficits. Paired reactivity at 39 and 73 kDa associated with increased irritability Reactivity to 39 kDa (alone or paired with 73 kDa) associated with BAP	Braunschweig et al., 2012a
37 ASD and TD mothers and their children ages 3-13 years	IHC for plasma reactivity to rhesus macaque brain tissue. Immunoblot reactivity to fetal and adult rhesus macaque brain proteins.	Reactivity at 37 and 73 kDa or 39 and 73 kDa found only in ASD mothers No significant differences in reactivity seen in ASD children vs. TD.	Rossi et al., 2013
Preschool aged males: 131 ASD (10 with 37/73 kDa IgG+ mothers) 50 TD, all negative for auto-Abs	MRI scan (during sleep) to evaluate total brain volume and compare maternal auto-Ab positive group to maternal auto-Ab negative groups	↑ abnormal brain enlargement in ASD, both groupsASD children with 37/73 kDa IgG+ mothers had more extreme abnormal brain enlargement compared to Ab negative ASD and TD groups, specifically in the frontal lobe.	Nordahl et al., 2013
246 ASD mothers 149 TD mothers	Plasma reactivity to fetal macaque brain verified by immunoblotting. Protein enrichment via PlasmPrep cell protein fractionation, 2-D electrophoresis and mass spectrometry.	6 brain proteins that has plasma reactivity were identified: lactate dehydrogenase A and B (LDH), cypin, stress-induced phosphoprotein 1 (STIP1), collapsin response mediator proteins 1 and 2 (CRMP1, CRMP2) and Y-box-binding protein (YBX1)Reactivity to any alone or in combination significantly was associated with ASD outcome ↑ stereotypical associated with reactivity to LDH, and combined reactivity to LDH/STIP1 or LDH/STIP1/CRMP1 ↑ overall impairment associated with reactivity to LDH and CRMP1	Braunschweig et al., 2013
2431 ASD mothers, 653 controls of child-bearing age	Plasma IHC reactivity to mouse brain	↑ presence of brain-reactive auto-Abs in ASD mothers compared to control women Presence of brain-reactive auto-Abs associated with anti-nuclear autoantibodies and increased prevalence of autoimmune diseases, especially RA and SLE.	Brimberg et al., 2013
333 ASD mothers 355 ASD 142 SIB	Child and mother plasma reactivity to Rhesus macaque brain tissue and human adult cerebellum by immune-blotting	Reactivity at 37, 39 and/or 73 kDa anti-brain auto-Abs associated with impaired language development, neurodevelopmental delay and sleep/wake cycle disturbances. Presence of the 62 kDa autoAb in the child associated with maternal reactivity at 39 and/or 73 kDa.Child results listed in Table 4.	Piras et al., 2014

*ASD, autism spectrum disorders; IHC, immunohistochemistry; TD, typically developing; kDa, kilodalton; GFAP, glial fibrillary acidic protein; MBP, myelin basic protein; DD, non-ASD developmentally delayed; PCR, polymerase chain reaction; PBMC, peripheral blood mononuclear cells; BAP, broader diagnosis of autism spectrum disorder; IgG, immunoglobulin G; MRI, magnetic resonance imaging; 2-D, two dimensional; auto-Abs, auto-antibodies; RA, rheumatoid arthritis; SLE, systemic lupus erythematosus; SIB, typically developing sibling*.

The first evidence of such antibodies was found in 2003 in the serum of a mother with two children on the autism spectrum. These antibodies were found to be reactive to Purkinje cells and other neuronal proteins in rodent brain tissue (Dalton et al., 2003). Since then, several studies have identified maternal antibodies with reactivity to various brain proteins of different molecular weights in mothers of ASD subjects. For example, Zimmerman et al. found multiple patterns of reactivity to rat brain proteins of low kilodalton (kDa) weight, and one at 250 kDa (Zimmerman et al., 2007). When measured by Western blot for reactivity to human and rodent fetal brain tissue, sera from ASD mothers had significant reactivity to a 36 kDa protein present in both human and rodent fetal brain and dense banding at 61 kDa (Singer et al., 2008). Braunschweig et al. observed immunoreactivity to proteins at approximately 37 and 73 kDa exclusively in the mothers of children with ASD, and found these to be associated with increased language deficits in the child. Additional reactivity to a pair of bands in the region of 39 and 73 kDa was associated with increased irritability and self-injurious behavior (Braunschweig et al., 2008, 2012a). Multiple studies have since confirmed the presence of maternal autoantibodies (MAbs) with paired reactivity to 37 and 73 kDA proteins exclusive to mothers of children with ASD (Croen et al., 2008; Nordahl et al., 2013; Rossi et al., 2013). Consistent with these studies, Piras et al. found that single (or combinations of) maternal anti-brain antibodies correlated with severity of language and other behavioral impairments, and that the presence of a specific autoantibody at 62 kDa in the child correlated with the presence of autoantibodies in the mother (Piras et al., 2014). Brimberg et al. confirmed that anti-brain antibodies are significantly more prevalent in mothers of children with ASD than typically developing children, and a majority of these women who harbor anti-brain antibodies also harbor anti-nuclear antibodies common to autoimmune disorders (Brimberg et al., 2013). Although the presence of these maternal autoantibodies were associated with risk of ASD in offspring, these studies are limited in that no clear mechanism was identified, which limits our understanding of how they might contribute to the etiology of ASD.

Animal models have allowed us to identify some pathogenicity of these autoantibodies (Table 3). Passive transfer of anti-brain antibodies from mothers of children with ASD to animals during gestation led to ASD-like pathology in both rodent (Singer et al., 2009; Braunschweig et al., 2012b) and primate offspring (Martin et al., 2008; Bauman et al., 2013), Target antigens to these maternal autoantibodies have since been identified as lactate dehydrogenase A and B (LDH−37 kDa band), cypin (previously undetected 44 kDa band), stress-induced phosphoprotein 1 (STIP1-upper 73 kDa band), collapsin response mediator proteins 1 and 2 (CRMP1/2-lower 70 kDa band) and Y-box-binding protein (YBX1–39 kDa band), with individual and combinations of maternal autoantibodies specific to these antigens increased in mothers of children with ASD. Increased stereotypical behavior and overall impairment were observed in children of mothers who possessed combinations of these autoantibodies (Braunschweig et al., 2013). Using structural magnetic resonance imaging (MRI), Nordahl et al. showed enlarged brain volume in male children born to mothers harboring anti-brain antibodies (Nordahl et al., 2013). In rodents, maternal autoantibodies administered during gestation were found to be able to migrate into the cortical parenchyma and alter coronal development by binding to radial glial cells in the ventricular zone and increased the number of neuronal precursor cells in the subventricular zone, increasing brain size and weight. Administration of autoantibodies also led to decreased numbers of mature dendritic spines in the adult cortex of mice, with STIP1 blockade being the likely culprit due to its importance in neuritogenesis, the sprouting of neurites that later develop into dendrites (Martinez-Cerdeno et al., 2016; Ariza et al., 2017). Mice exposed to maternal autoantibodies during the embryonic stage displayed ASD-like behaviors including increased repetitive behaviors and altered social interactions (Camacho et al., 2014). Generation of endogenous autoantibodies prior to gestation to the fetal brain epitopes identified in ASD mothers also led to social deficits and increased repetitive grooming in adult offspring mice (Jones et al., 2018).

**Table 3:** Preclinical studies of maternal autoantibodies and ASD-like pathology.

**Pre-clinical model details**	**Summarized findings**	**References**
**Mouse model**
Sera from 1 ASD mother (auto-Ab+) and 4 TD mothers injected into pregnant MF1 mice at varied time points. Offspring behaviors and cerebellar chemistry measured with standard behavioral tests and MRS	ASD-sera exposed offspring exhibited: ↓ reflexes ↓ exploration ↓ spatial orientation ↓ creatine and choline concentrations in cerebellum No impairments in memory	Dalton et al., 2003
**Non-human primate model** Purified IgG from separately pooled from 21 ASD and 7 TD maternal sera, measured for presence of auto-Abs. 4 Rhesus macaques were injected IV with auto-Ab+ IgG from ASD maternal sera and 4 were injected with auto-Ab- IgG from TD maternal sera at GD 27, 41, and 55. Behaviors assessed at preweaning and postweaning time points.	ASD-IgG exposed offspring exhibited: ↑ whole-body stereotypies ↑hyperactivity No significant differences seen in social behaviors	Martin et al., 2008
**Mouse model** Purified IgG from separately pooled from 63 ASD and 63 TD maternal sera, injected IP into 26 pregnant C57Bl/6J mice total, 13 dams per group, at E13-E18. Offspring from untreated and from saline injected mice included as negative controls. Several behavioral and neurodevelopmental outcomes measured.	ASD-IgG exposed offspring exhibited: ↑ anxiety, startle, and hyperactivity ↑ Iba1 staining indicating microglia activation in E18 embryos ↑ BDNF at adolescence Social deficits seen at adulthood IL-12 detectable in E16 embryos	Singer et al., 2009
**Mouse model** Purified IgG from pooled from 3 ASD maternal sera positive for 37 kDa and 73 kDa fetal brain protein reactivity. Purified IgG also pooled from 3 TD maternal sera absent of reactivity. Single IV injection of purified IgG per group given to pregnant C57Bl/6J mice at GD 12, saline given as negative control. Offspring assessed for behavioral abnormalities and alterations in neurodevelopment.	ASD-IgG exposed offspring exhibited: ↓ weight and body length ↓ sensory and motor development prior to weaning ↑ anxiety ↑ vocalizations during separation-induced stress in males at PND8 Social deficits trended in males but did not reach significance No differences seen in stereotypical behaviors No differenced seen in numbers of CA1 hippocampal neurons	Braunschweig et al., 2012b
**Non-human primate model** Purified IgG from ASD maternal sera positive for 37 kDa and 73 kDa autoAbs and purified IgG from TD maternal sera absent of reactivity injected IV into two groups of pregnant Rhesus macaques at GD 30, 44, 58, 72, 86, and 100.	ASD-IgG exposed offspring exhibited: ↑ maternal protectiveness during pre-weaning stage ↑ inappropriate and frequency of social approach in juveniles ↑ brain volume in males compared with controls, mainly white matter and most profoundly in the frontal lobes.	Bauman et al., 2013
**Mouse model** Purified IgG from ASD maternal sera positive for autoAbs or from TD maternal sera absent of reactivity was injected directly into cerebral ventricles of E14 embryonic Swiss Webster mice. Behaviors measured with battery of behavioral assays	ASD-IgG injected adult offspring exhibited: ↑ grooming ↑ marble burying No differences in social approach, however ASD-IgG mice had↑ time spent with novel object compared to mice injected with TD-IgG	Camacho et al., 2014
**Mouse model** Biotinylated IgG from ASD maternal sera positive for 37 kDa and 73 kDa fetal brain protein reactivity or from TD maternal sera absent of reactivity was injected directly into cerebral ventricles of E14 or E16 embryonic Swiss Webster mice. Brain reactivity and quantification assessed with IHC and stereology.	Brain from ASD-IgG injected embryos exhibited: Reactivity to RG cells (neural stem cells) in VZ ↑proliferative Pax6+ RG cells in the SVZ ↑mitotic precursor cells in the SVZ of the ganglionic eminence (a neurodevelopmental structure that guides cell and axon migration) RG cells translocated much earlier than control mice ↑ brain weight and rostro-caudal length ↑somal volume in neurons	Martinez-Cerdeno et al., 2016
**Mouse model** Purified IgG from ASD maternal sera positive for 37 kDa and 73 kDa fetal brain protein reactivity or from TD maternal sera absent of reactivity was injected directly into cerebral ventricles of E14 embryonic Swiss Webster mice. Changes in dendritic arbor and spine population assessed with Golgi method and Neurolucida.	Adult brain from ASD-IgG injected embryos showed: ↓ length and volume of dendritic spines on neurons of the frontal cortex ↓ total number of spines on neurons in frontal cortex ↓ total number of spines on neurons in occipital cortex ↓ spine density of apical dendrites, and ↓ number of mature spines on basal and apical dendrites in occipital cortex	Ariza et al., 2017
**Mouse model** Mixtures of 21 synthesized epitopes of LDH-A, LDH-B, STIP1, and CRMP1 (fetal brain target peptides) plus adjuvant were injected SC 5 times into dams prior to mating (MAR-ASD). Control females injected SC with saline plus adjuvant. Maternal sera tested for verification of endogenous autoAb production. Offspring behaviors measured with behavioral assays	Epitope-specific antibodies were successfully produced and persisted in dams through end of lactation MAR-ASD offspring exhibited: ↑ weight and head width ↑ repetitive behaviors ↓ social behaviors, including male-female social interactions	Jones et al., 2018

*ASD, autism spectrum disorder; autoAb, autoantibody; MRS, magnetic resonance spectroscopy; IgG, immunoglobulin G; TD, typically developing; IV, intravenous; GD, gestational day; IP, intraperitoneal; Iba1, Ionized calcium binding adaptor molecule 1; E13, embryonic day 13; BDNF, brain derived neurotrophic factor; IL-12, interleukin-12; IHC, immunohistochemistry; RG cells, radial glial cells; VZ, ventricular zone; SVZ, subventricular zone; LDH, lactate dehydrogenase; STIP1, stress-induced phosphoprotein 1; CRMP1, collapsin response mediator protein 1; SC, subcutaneous*.

Although the origin(s) of these autoantibodies are unknown, they are more frequent in mothers carrying a functional variant of the Met Receptor Tyrosine Kinase (*MET*) promoter which leads to reduced production of MET receptor tyrosine kinase, a receptor involved in immune regulation (Heuer et al., 2011). Mothers carrying the variant *MET* allele had reduced interleukin (IL)-10, an important regulatory cytokine, suggesting that dysfunction in immune regulation may be driving the production of autoantibodies (Heuer et al., 2011). Understanding the development of these MAbs and the role of the proteins they target in neurodevelopment is important due to the substantial number of ASD cases found to involve these antibodies. Research in this area may lead to diagnostic tools that assess maternal risk, as well as possible treatments and early interventions for children with maternal autoantibody-related ASD.

### Maternal immune activation (MIA)

Maternal infection during pregnancy has been implicated as a potential environmental risk factor for ASD (reviewed in Patterson, 2011). In case series reports, infections during pregnancy, such as rubella, measles or toxoplasmosis, can negatively impact early neurodevelopment of the fetus. In population based studies, viral, and bacterial infections occurring in the first or third trimester, respectively, or maternal fever during gestation pose an increased risk for later development of ASD in offspring (Atladottir et al., 2010; Zerbo et al., 2013). Both rodent and non-human primate models of maternal infection during gestation have supported epidemiological studies, showing alterations in ASD-associated behaviors and immune dysregulation that persisted into adulthood in offspring born to mothers exposed to viral or bacterial antigens during gestation (Schwartzer et al., 2013; Bauman et al., 2014; Meyer, 2014; Onore et al., 2014; Choi et al., 2016; Rose et al., 2017). Maternal asthma during pregnancy has also previously been linked with ASD (Croen et al., 2005; Lyall et al., 2014). Furthermore, in a cohort of 220 children with ASD, those whose mothers had a history of allergies or asthma during pregnancy displayed more severe social impairments (Patel et al., 2017). Animal models of maternal gestational asthma have validated both behavioral and immune abnormalities in offspring, including epigenetic alterations in methylation of immune pathway genes in microglia–the resident immune cells of the brain (Schwartzer et al., 2015, 2017; Vogel Ciernia et al., 2018).

**Figure 1:**
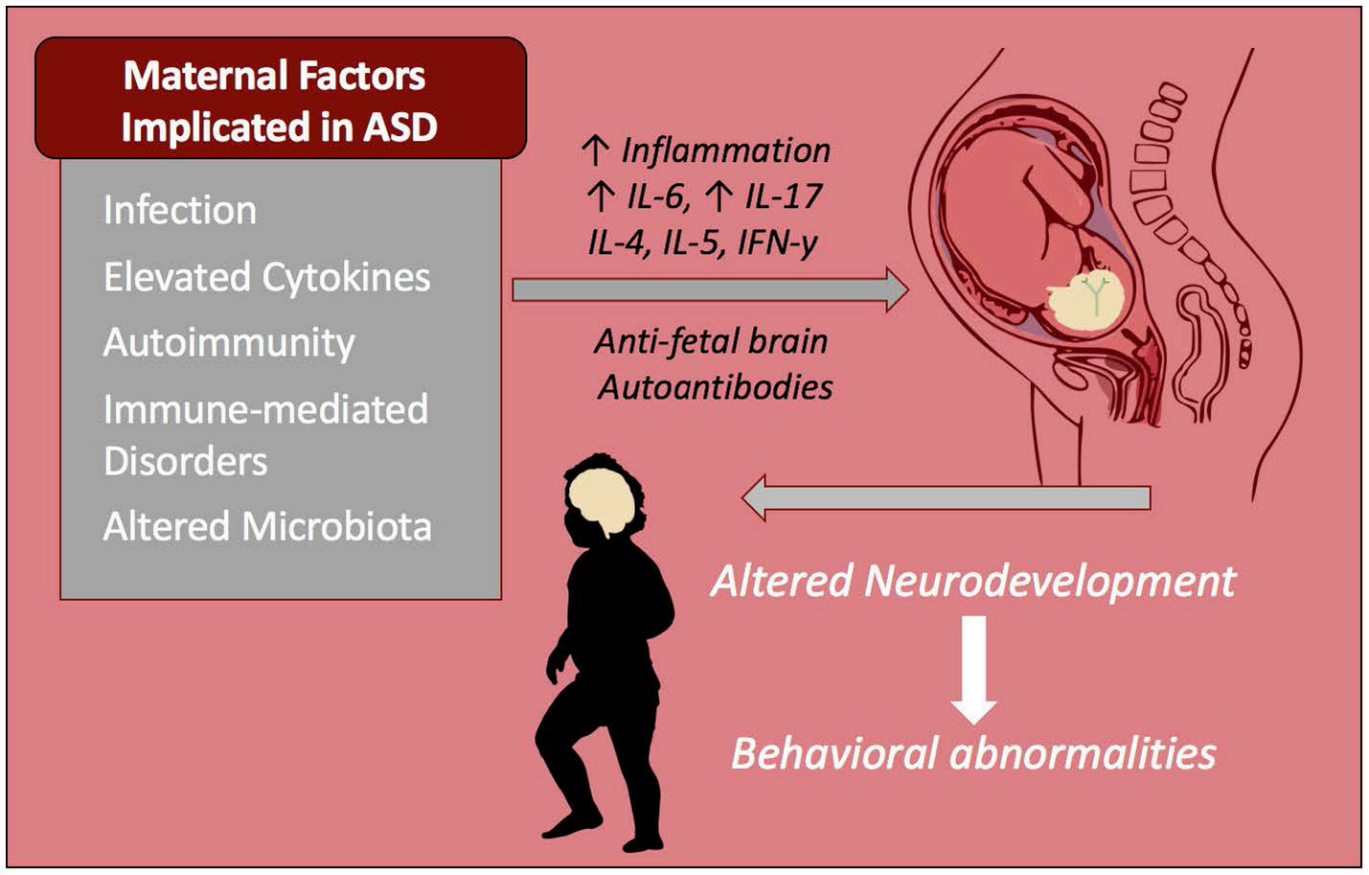
Maternal immune influences during gestation increase risk of ASD. Infection and immune-mediated/autoimmune disorders in the mother are known risk factors that increase the chances of a child developing ASD. These inflammatory factors as well as altered maternal microbiota may be contributing to increased inflammatory cytokines and/or autoantibodies that react to fetal brain tissue. These factors alter the immune profile and neurodevelopment of the child and are linked to behavioral abnormalities seen in ASD including repetitive behaviors, stereotypies, anxiety, and impaired social behaviors.

The driving mediators of MIA-associated ASD pathology are most likely elevations in maternal cytokines and chemokines. In addition to their roles as immune-mediators, these signaling proteins play important roles in central nervous system (CNS) development and are involved in migration of neuronal precursors, neuronal maintenance, synaptic pruning and plasticity, thus they need to be tightly regulated (Deverman and Patterson, 2009). Cytokines that cross the placenta, such as IL-6 and IL-4, have the potential to alter epigenetic regulation of gene transcription (Nardone and Elliott, 2016). Elevations of cytokines and chemokines in both maternal serum during gestation and amniotic fluid are associated with increased risk of ASD in human subjects (Goines P. E. et al., 2011; Abdallah et al., 2012; Jones et al., 2016). Mechanistically, maternal cytokines such as IL-6 and IL-17 may be mediating inflammation either at the placenta or directly in the developing fetal brain (Smith et al., 2007; Hsiao et al., 2012; Choi et al., 2016). Moreover, it is possible that maternal inflammation may be contributing to the development of maternal autoantibodies (Figure 1). As well as cytokine driven responses, other downstream events occur that can effect immune and neuronal development. For instance, in the LPS model of MIA trace metal levels are altered included the sequestration of zinc in both the dams and offspring (Coyle et al., 2009; Kirsten et al., 2015; Kirsten and Bernardi, 2017).

Recent evidence in a mouse model of MIA suggest that maternal microbiota composition may be driving maternal inflammation to skew toward IL-17 production, inducing behavioral changes. Dams lacking certain microbiota did not produce IL-17 and their offspring did not exhibit aberrant behavioral phenotypes. IL-17 blockade was also effective in preventing behavioral abnormalities (Choi et al., 2016; Kim et al., 2017). This is a notable finding considering our understanding of IL-17 as a central driver of autoimmune disorders (Zhu and Qian, 2012). A better understanding of the role of IL-17 signaling during gestation and within the fetal brain may hopefully lead to therapeutics targeting this cytokine.

## Immune findings in individuals with ASD

### Immune mediated co-morbidities

**Figure 2:**
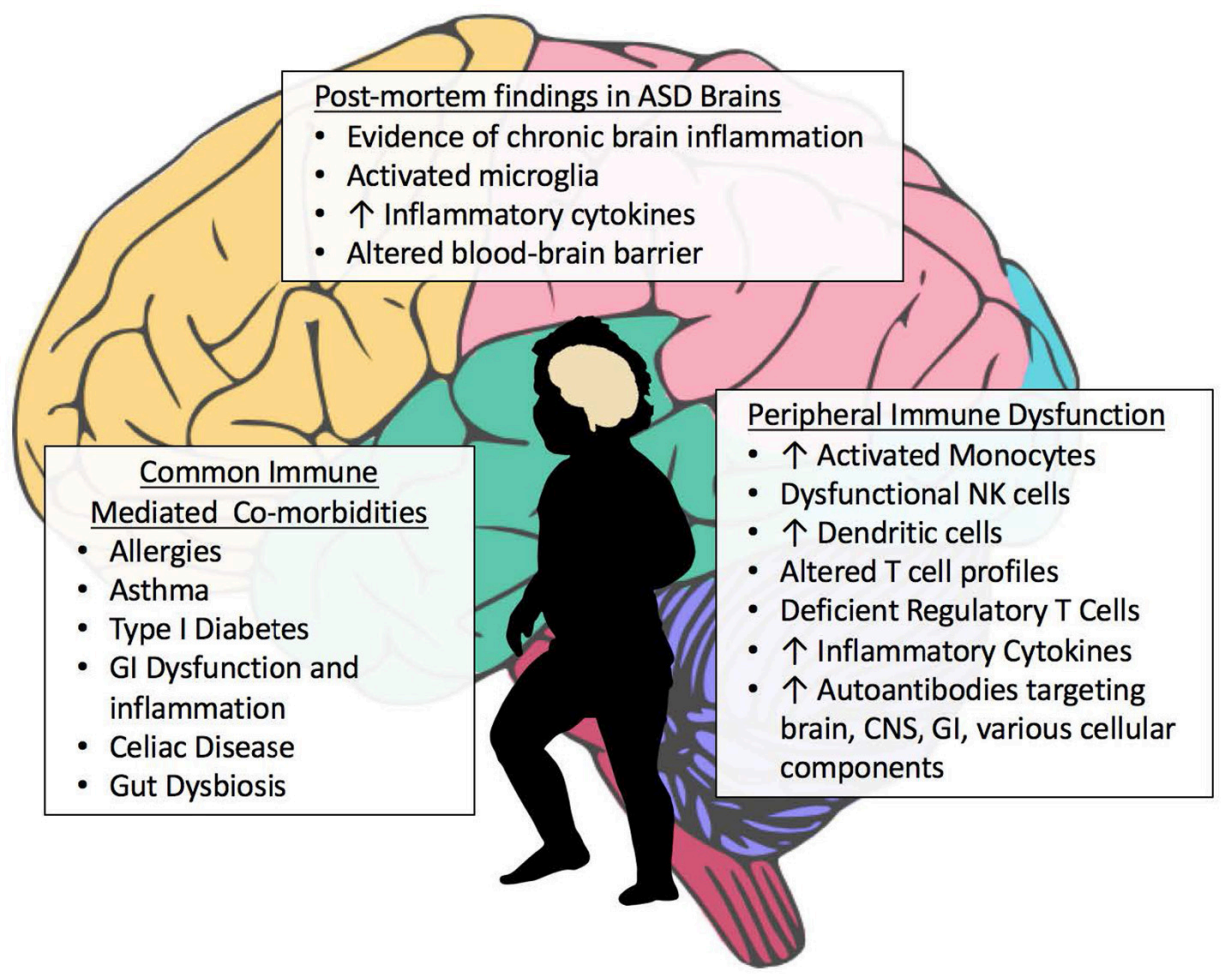
Aberrant Immune Findings in Individuals with ASD. Evidence of immune dysfunction in ASD has grown substantially in recent years. Individuals with ASD commonly have immune-mediated comorbidities such as allergies and gastrointestinal (GI) dysfunction that may be contributing to the aberrant behaviors seen in ASD. Although research in this area is occasionally contradictory, the vast majority of immune studies in individuals with ASD have shown immune dysfunction and dysregulation. Several studies have found elevations in inflammatory cells and cytokines, both peripheral as well as within post-mortem brain tissue. A variety of autoantibodies targeting various tissues and cellular components throughout the body have been identified in subsets of subjects with ASD. Individuals with ASD also have fewer regulatory T cells.

Among the many immune findings in ASD, several recent large-scale studies have indicated that individuals with ASD have frequent immune-mediated comorbid health issues that may progress or predispose to later-life autoimmune conditions (Figure 2). Zerbo and colleagues found that allergies and autoimmunity diagnoses were significantly more common in children with ASD, with odds ratios of 1.22 and 1.36, respectively (Zerbo et al., 2015). Children with ASD, as surveyed in the National Health Interview Survey, require higher health care use and have a higher prevalence of most medical conditions defined in autoimmune areas, compared to those without developmental disabilities (Schieve et al., 2012). However, as many autoimmune conditions do not manifest until adulthood, the young age of most study populations is a limitation to finding associations between ASD and autoimmunity. To determine significant pathogenic components of ASD, cluster analysis found immune dysfunction to be the best-defined cluster for ASD. This study noted that immune dysfunction underlined the majority of comorbidities observed in ASD (Sacco et al., 2012). Allergic diseases are overrepresented in ASD, and in some individuals may influence behaviors and severity of core behavioral deficits (Mostafa et al., 2008; Shibata et al., 2013). A large epidemiological study found that asthma was 35% more common in children with ASD compared to typically developing children (Kotey et al., 2014). This supports a previous study that found a significantly increased risk of asthma in ASD subjects, with an odds ratio of 1.74 (Chen et al., 2013). Increased risk of type 1 diabetes, allergic rhinitis, atopic dermatitis, urticaria and a trend toward increasing comorbidity with Crohn's disease are also observed in subjects with ASD (Chen et al., 2013). The same group looked specifically at asthma in a nationwide population-based prospective study over 8 years and found children with asthma in early life had an increased risk of developing ASD (adjusted hazard ratio: 2.01, 95% confidence interval: 1.19–3.40) (Tsai et al., 2014). An analysis of over 1500 adults with ASD showed significantly increased rates of medical conditions in individuals with ASD compared to non-ASD controls, including but not limited to immune co-morbidities, gastrointestinal (GI) disorders, diabetes, obesity, seizures, and sleep disorders (Croen et al., 2015).

One of the most commonly reported comorbidities in ASD is the incidence of GI dysfunction and inflammation in the GI tract (Figure 2). Assessment for GI dysfunction is often challenging in individuals with ASD due to communication deficits. Reported prevalence of GI disturbances varies widely, with ranges from 9 to over 90% in ASD subjects; however, the 2013 Interagency Autism Coordinating Committee concluded that at least 50% of children with ASD had GI issues (discussed in Mcelhanon et al., 2014). A 2014 assessment of 960 children enrolled in the Childhood Autism Risks from Genetics and the Environment (CHARGE) study found that children with ASD had significantly increased odds of having at least one GI symptom compared to typically developing controls (OR 7.92 [4.89–12.85]) (Chaidez et al., 2014). Discrepancies in prevalence rates of GI comorbidities in ASD are likely due to differences in defining criteria for symptoms, referral bias, variations in samples sizes and sources of data, as well as the timing in which symptoms were reported (Buie et al., 2010; Coury et al., 2012). Early studies examining intestinal dysfunction in ASD showed increased monocytes, lymphocytes, NK cells, eosinophils, and intraepithelial lymphocytes in duodenal biopsies, and autoantibody IgG and co-localized C1q complement bound to the basal membrane of GI epithelial cells (Torrente et al., 2002). This autoimmune phenomena—directed against the gut lumen barrier—could be responsible for the increased intestinal permeability seen in individuals with ASD (de Magistris et al., 2010). Inflammatory immune cells were found to infiltrate the epithelium and lamina propria of the GI tract (Furlano et al., 2001; Torrente et al., 2002; Ashwood et al., 2003). Further, studies showing increased production of inflammatory cytokines and decreased regulatory IL-10 production by mucosal Cluster of Differentiation(CD)3^+^ T cells suggested the presence of increased inflammation and dysregulation of mucosal immune responses that could be contributing to disruption of the intestinal barrier (Ashwood et al., 2004; Ashwood and Wakefield, 2006). Mucosal gene profiling adds further support to these findings, with upregulation of cytokine production, including IL-17 and increased immune activation in children with ASD who have comorbid GI issues (Walker et al., 2013).

Increased inflammation and dysregulation of the GI tract in ASD is important as this compartment comprises a significant percentage of immune cells in the body, and immune cells educated here participate in immune function throughout the body. Cellular education and the maintenance of regulatory immune cells in the mucosal GI compartment are strongly influenced by the community of microbes that reside here, and this influences systemic immune homeostasis (reviewed in Wu and Wu, 2012). Additionally, if the intestinal barrier is disrupted, antigenic material from the lumen of the gut may enter the periphery and initiate an immune response. Indeed, ASD subjects with GI comorbidities have increased peripheral inflammation and lower production of regulatory cytokines (Jyonouchi et al., 2001, 2005, 2011; Ashwood and Wakefield, 2006; Rose et al., 2018). Circulating antigens and bacterial metabolites could also directly influence the brain if both the intestinal and blood-brain barriers are not sufficiently intact. This is one proposed mechanism of how the microbiota-gut-brain axis may have pathological involvement in neurological disorders (Cryan and Dinan, 2012). Interestingly, recent findings in post-mortem tissue suggest that individuals with ASD have alterations in the blood-brain barrier and deficiencies in gene expression of intestinal tight junction proteins (Figure 2) (Fiorentino et al., 2016). Several studies have also indicated that individuals with ASD have dysbiotic alterations in gut flora and altered bacterial metabolites (Figure 2) (Finegold et al., 2010; Williams et al., 2011, 2012; Gondalia et al., 2012; Kang et al., 2013; Tomova et al., 2015; Strati et al., 2017; Hughes et al., 2018), including recent findings that children with ASD who exhibit GI symptoms have differences in microbiota compared to children with ASD who have no GI dysfunction. Their microbiota also differed from typically developing children with similar GI symptoms (Rose et al., 2018). These studies suggest an ineffective immune response to bacteria in ASD, or production of inflammatory mediators leading to preferential bacterial growth (Spees et al., 2013). It is unclear whether the dysbiosis seen in ASD is driving the immune dysfunction and altered immune regulation, or is a result of it and much more research is needed in this area.

It has been suggested that food allergy may play a role in GI dysfunction, and some parents of children with ASD have seen behavioral improvements after implementing diets that eliminate suspect antigens such as gluten and casein; however, the role of food allergies remains controversial. Reports of IgE-mediated food allergies correlating with GI symptoms are inconclusive (Buie et al., 2010), although, one group found a high incidence of non-IgE-mediated food allergy in younger children with ASD (Jyonouchi et al., 2008). Celiac disease has also been reported to have an association with ASD (Barcia et al., 2008; Ludvigsson et al., 2013). A recent Italian study looked at a cohort of 382 preschoolers diagnosed with ASD, and found the prevalence of celiac disease among the ASD cohort was significantly increased compared to the general pediatric population, with an overall prevalence of 2.62% (Calderoni et al., 2016). This supports the earlier work by Barcia et al. who found a 3.3% prevalence of celiac disease in ASD by exploring biopsied tissue (Barcia et al., 2008). As GI disturbances may exacerbate behavioral symptoms, better screening techniques that include identification of behaviors associated with GI distress may help to better elucidate the actual prevalence within this population.

Additional research seeking to identify a genetic and molecular basis for the comorbidities plaguing individuals with ASD consistently found dysregulation of multiple innate signaling pathways by utilizing searches of curated gene pathways. Nine Kyoto Encyclopedia of Genes (KEGG) pathways were recently identified to overlap in ASD and other disease comorbidities common in ASD. For example, three pathways involved in regulation of the immune response, the toll-like receptor (TLR), nucleotide-binding and oligomerization domain (NOD) pathways, and chemokine signaling pathways significantly overlapped with asthma and inflammatory bowel disease (Nazeen et al., 2016). Further studies are needed to examine these pathways in ASD. However, it is important to note that not all of these dysregulated pathways may exist in individuals with ASD (Campbell et al., 2013), but may be dysregulated in those with immune comorbidities and help account for the wide heterogeneity and conflicting results seen in some studies in ASD.

### Autoantibodies in individuals with ASD

In addition to antibodies targeting the GI epithelium, autoantibodies specific to self-proteins in the brain, CNS and cellular components have been frequently reported in individuals with ASD (Figure 2, Table 4). Autoantibodies are a common feature in autoimmunity, and their presence may be predictive of the development of certain autoimmune disorders (Anaya et al., 2007; Lleo et al., 2010). Presence of autoantibodies that react to components of the brain and CNS in individuals with ASD have been identified since as early as 1988, when antibodies to neuron-axon filament proteins (NAFP) were found in 10 out of 15 children with ASD (Singh et al., 1988). A year later, researchers identified IgG and IgM antibodies that target cerebellar neurofilaments (Plioplys et al., 1989). Anti-myelin basic protein (MBP) antibodies were identified in individuals with ASD in 1993 (Singh et al., 1993) and later supported by additional studies (Singh et al., 1998; Connolly et al., 2006). The anti-MBP results have been replicated in additional studies, including a 2013 investigation that linked these autoantibodies to both severity of ASD as well as allergic manifestations (Mostafa and Al-Ayadhi, 2013). However, other studies have refuted these findings which underscores the wide variations of immune phenotypes seen in ASD (Libbey et al., 2008).

**Table 4:** Studies identifying presence of autoantibodies in individuals with ASD.

**Subject details**	**Methods**	**Summarized findings**	**References**
48 autism (5.9 ± 3.9 years) 19 CDD (7.0 ± 2.4 years) 14 PDD-NOS (4.8 ± 3.9 years) 9 LKS (7.4 ± 2.3 years) 37 epilepsy (5.9 ± 3.8 years) 29 HC (4.3 ± 2.0 years) 21 NNI (4.2 ± 2.6 years)	Serum ELISA measurements of BDNF, IgG/IgM auto-Abs to BDNF, endothelial cells, MBP, and histones *Note: Subject numbers indicate total included in study. Actual numbers varied slightly depending on assay*	↑ BDNF in ASD, CDD compared to HC and NNI ↑ anti-BDNF IgM and IgG in autism, CDD and epilepsy compared to HC ↑ IgM to endothelial cells in autism, CDD, PDD-NOS, and epilepsy compared to HC and NNI ↑ IgG to endothelial cells in autism and PDD-NOS compared to HC ↑ IgM and IgG to MBP in autism, CDD, PDD-NOS, and epilepsy compared to both HC and NNI, LKS not elevated	Connolly et al., 2006
29 ASD (3–12 years) 9 SIB (4–8 years) 13 TD (9–17 years)	Serum ELISA and Western blot reactivity to human brain	↑ reactivity to 100 kDa epitope in caudate putamen and prefrontal cortex in ASD ↑reactivity to 73 kDa epitope in cerebellum and cingulate gyrus in ASD and SIB	Singer et al., 2006
63 ASD (2–15 years) 63 TD (2–14 years) 25 SIB (1–13 years) 21 DD (2–5 years)	Western blot of plasma reactivity to adult human hypothalamus and thalamus protein extracts	↑ reactivity to 52 kDA thalamus and hypothalamus proteins in ASD ↑ reactivity to 3 hypothalamus proteins (42–48 kDa MW)	Cabanlit et al., 2007
11 ASD 9 SIB (>6 years)	72-h neuronal culture analyzed for effect of ASD sera on differentiation of NPCs by immunoblotting, morphometry, and immunocytochemistry	Treatment with ASD sera: ↓ NPC proliferation ↑ cell migration ↑small cells with processes ↑ length and number of processes ↑ synaptogenesis	Mazur-Kolecka et al., 2007
33 ASD (7.3 ± 3.0 years) 26 regressive autism (6.7 ± 2.7 years) 25 TD (8.9 ± 3.4 years) 24 Tourette syndrome (10.0 ± 2.6 years)	Plasma ELISA and Western blot reactivity to MBP	↑ auto-Abs to MBP found in regressive autism compared to classic (infantile) autism and Tourette syndrome subjects.	Libbey et al., 2008
63 ASD (2–15 years) 63 TD (2–14 years) 25 SIB (1–13 years) 21 DD (2–5 years)	Western blot of plasma reactivity to human cerebellar protein extracts. Cerebellar-specific auto-Abs detected by IHC of *Macaca fascicularis* monkey cerebellum.	↑auto-Abs to 52 kDa cerebellar protein in ASD ↑“intense immunoreactivity” to Golgi cells of the cerebellum in ASD, associated with auto-Abs to 52 kDa cerebellar protein	Wills et al., 2009
37 ASD (1–12 years) 37 TD (1–14 years)	Measured effect of ASD sera on cell response to oxidative stress via immunoblotting, morphology, immunofluorescence, apoptosis, and proliferation assays.	Oxidative stress reduced proliferation in differentiating NPCs treated with TD sera. Effect was not as prominent with ASD sera, indicating an altered response to oxidative stress.	Mazur-Kolecka et al., 2009
20 ASD (3.0 ± 0.4 years) 12 TD (3.0 ± 1.2 years)	Taqman Real time PCR to detect serum mtDNA. Serum ELISA analysis to detect mtDNA antibodies	↑ extracellular mtDNA in ASD ↑ anti-mtDNA auto-Abs (type 2) in ASD	Zhang et al., 2010
277 ASD (70 BAP) 189 TD (2–5 years)	Western blot for child plasma reactivity to Rhesus macaque cerebellum protein medley	↑auto-Abs to 45 kDa protein in ASD ↑auto-Abs to 62 kDa protein in BAP Increases in either auto-Ab was associated with lower adaptive and cognitive scores, increased aberrant behaviors.	Goines P. et al., 2011
54 ASD 54 TD (4–11 years)	Serum anti-ganglioside M1 Abs were measured by ELISA	↑ antiganglioside M1 auto-Abs in ASD, especially in severe compared to mild or moderate autism.	Mostafa and Al-Ayadhi, 2011
86 ASD (2.0–5.6 years) 43 TD (2.3–4.7 years)	IHC for plasma reactivity to sections of macaque monkey brain (methods similar to Wills et al., 2009), results compared to behavioral assessments.	No differences in rate of plasma immunoreactivity to cerebellar Golgi neurons and other neural elements in ASD vs. TD, however immunoreactivity associated with worsening behavior and higher multiple CBCL scores.	Rossi et al., 2011
7 ASD with reactivity (2.5 to 7 years) 7 ASD with no reactivity 6 TD with no reactivity (2.5 to 8 years)	IHC: follow up of subgroup of ASD children from previous study (Wills et al., 2009) with reactivity to cerebellar 52-kDa protein and to Golgi cell region of the cerebellum. IHC to detect plasma immunoreactivity in the maqaque and male mouse brains.	Reactivity seen in previous study identified as GABAergic interneurons (based on co-localization of staining to calcium-binding proteins). Reactivity extended to other regions of the brain with slight preponderance to superficial layers of the cortex.	Wills et al., 2011
80 ASD 80 TD (6–12 years)	Indirect immunofluorescence used to measure serum anti-neuronal antibodies	↑ anti-neuronal auto-Abs in ASD, associated with increased severity of autism and seen more frequently in females ASD (90 vs. 53.3%, *P* = 0.001).	Mostafa and Al-Ayadhi, 2012b
50 ASD 30 TD (5–12 years)	Serum ELISA measurements of 25-hydroxy vitamin D and anti-MAG autoAbs	↓ 25-hydroxy vitamin D in ASD ↑ anti-MAG auto-Abs in ASD 25-hydroxy vitamin D levels negatively correlated with CARS scores and anti-MAG auto-Abs	Mostafa and Al-Ayadhi, 2012a
54 ASD 22 DD 33 TD (2–5 years)	Plasma ELISA measurements of anti-cardiolipin, anti-phosphoserine, and anti-β-glycoprotein 1 auto-Abs	↑ auto-Abs to cardiolipin, phosphoserine, and β-glycoprotein 1 in ASD compared to TD and DD controls, significantly associated with worsening behaviors.	Careaga et al., 2013
42 ASD 42 TD (6–11 years)	Serum ELISA measurement of human anti-MBP Abs. Severity of ASD and manifestation of allergic/asthma symptoms compared to results.	↑ auto-Abs to MBP and MAG in ASD, regardless of allergies. Severity of autism was also found to be associated with increased allergies.	Mostafa and Al-Ayadhi, 2013
93 ASD (2.9–17.4 years)	Patented process of identifying FRA: incubated serum with folate receptors then added radio-labeled folic acid. HPLC measurement of 5-MTHF in the CSF.	↑ prevalence of FRA in ASD sera. Blocking FRA correlated with CSF 5-MTHF concentrations in 16 children. Treatment with folinic acid improved attention, language and communication, and repetitive behaviors, with “moderate to much” improvement seen in 1/3 of children treated.	Frye et al., 2013
75 ASD (2–22 years) 30 DD (1–18 years)	Patented process of identifying FRA: incubated serum with folate receptors then added radio-labeled folic acid.	↑ prevalence of FRA in ASD vs. DD ↑ prevalence of FRA in parents of ASD vs. DD, suggesting familial autoimmune component to ASD	Ramaekers et al., 2013
20 ASD (1.4–5 years) 18 TD (1.4–4.4 years)	Immunoblotting and immunocytochemistry to detect serum auto-Abs against differentiating NPCs	↑ auto-Abs against human neuronal progenitor cell proteins of 55, 105, 150, and 210 kDa molecular weights in ASD subjects compared to controls. Strongest reactivity noted in NPCs expressing Tuj1.	Mazur-Kolecka et al., 2014
100 ASD 100 TD (4–11 years)	ELISA measurement of serum anti-ds-DNA Abs. Immunofluorescence measurement of serum antinuclear Abs.	↑ anti-ds-DNA and anti-nuclear auto-Abs in ASD Presence of anti-ds-DNA auto-Abs positively associated with a family history of autoimmunity.	Mostafa et al., 2014
355 ASD 142 SIB (2–47 years, mean age: 9.06)	Western blot plasma reactivity to homogenized Rhesus macaque brain tissue and human adult cerebellum	Plasma reactivity at 45 and 62 kDa brain proteins associated with autism severity and larger head circumference. 45 kDa reactivity associated with cognitive impairment/lower VABS scores while 62 kDa reactivity associated with stereotypies.	Piras et al., 2014
60 ASD 60 TD (3–12 years)	ELISA measurement of serum anti-nucleosome-specific antibodies.	↑ anti-nucleosome-specific auto-Abs in ASD, associated with family history of autoimmunity.	Al-Ayadhi and Mostafa, 2014
55 ASD (3–12 years) 25 TD (4–12 years)	ELISA measurement of plasma levels of anti-endothelial cell antibodies	↑anti-endothelial cell auto-Abs in children with autism compared to healthy controls, associated with autism severity.	Bashir and Al-Ayadhi, 2015
62 ASD (4–11 years) 62 TD (5–12 years)	ELISA measurement of serum ENA-78 and anti-neuronal auto-antibodies	↑ anti-neuronal auto-Abs in ASD ↑ENA-78 (neutrophil-recruiting chemokine CXCL5) associated with increases in anti-neuronal auto-Abs	Mostafa and Al-Ayadhi, 2015
40 ASD/FRAA-(7.0 ± 3.3 years) 16 ASD/FRAA blocking + (6.4 ± 3.0 years) 48 ASD/FRAA binding + (7.3 ± 3.1 years)	Measured redox, methylation, vitamins and immune biomarkers using various assays and compared to behavioral assessments	↓ 3-Chlorotyrosine (a marker of inflammation) was in those positive for blocking FRAs Presence of blocking FRAs in ASD associated with less severe ASD symptoms compared to ASD negative for these FRAs	Frye et al., 2016

*ASD, autism spectrum disorders; CDD, childhood disintegrative disorder with regression after age 2 years; PDD-NOS, pervasive developmental disorder-not otherwise specified; LKS, Landau-Kleffner syndrome; HC, healthy children; NNI, children with non-neurologic illnesses; ELISA, enzyme-linked immunosorbent assay; BDNF, brain-derived neurotrophic factor; MBP, myelin basic protein; SIB, typically-developing sibling; TD, typically developing child; kDa, kilodalton; DD, non-ASD developmentally delayed; NPCs, human neuronal progenitor cells; MEF, myelin-enriched fraction of the brain; AEF, axolemma-enriched fraction of the brain; IHC, immunohistochemistry; auto-Abs, autoantibodies; PCR, polymerase chain reaction; mtDNA, mitochondrial DNA; BAP, broader diagnosis of autism spectrum disorder; CBCL, The Child Behavior Checklist; MAG, myelin-associated glycoprotein; FRA, folate receptor antibodies; 5-MTHF, 5-methyltetrahydrofolate; CSF, cerebrospinal fluid; Tuj1, neuron-specific Class III β-tubulin; ds-DNA, double-stranded DNA; VABS, Vineland Adaptive Behavior Scales; ENA-78, Epithelial cell-derived neutrophil-activating peptide-78; CXCL5, C-X-C motif chemokine 5*.

More recent studies have found autoantibodies to the prefrontal cortex, caudate, putamen, cerebellum and cingulate gyrus regions of the brain (Singer et al., 2006) and hypothalamus (Cabanlit et al., 2007) in children with ASD. In 2009, researchers found that 21% of plasma samples from children with ASD had intense immunoreactivity to Golgi neurons in primate cerebellum, with no reactivity occurring in controls. These ASD autoantibodies reacted to a protein of a molecular weight of 52kDa in human cerebellum (Wills et al., 2009). A follow-up study identified reactivity to interneurons in other regions of the brain, including those in the superficial layers of the cortex. The target neurons were identified as specifically GABAergic. GABAergic Golgi neurons and interneurons are inhibitory, utilizing the neurotransmitter gamma-aminobutyric acid (GABA) to modulate nearby excitatory synapses. It is unknown whether these antibodies are able to cross the blood-brain barrier (Wills et al., 2011). However, if they are able to enter the brain and reach their target antigens, this could potentially alter numbers or activity of inhibitory neurons, and contribute to the imbalance in excitatory/inhibitory activity that has long been suggested to contribute to certain aspects of ASD (Rubenstein and Merzenich, 2003).

Eighty-six children with ASD and forty-three typically developing controls from the Autism Phenome Project, a large multidisciplinary study conducted at the MIND Institute, were further assessed for these neuronal autoantibodies. Similar reactivity to cerebellar Golgi neurons and interneurons was found throughout the brain in some children with ASD; however, the results were not significantly different than controls, and in contrast to (Wills et al., 2009), some typically developing children also exhibited positive staining. Although these results did not support previous findings that these autoantibodies occur solely in ASD, this group did find a correlation between immunoreactivity and increased scores on the Child Behavior Checklist (CBCL), indicating worsening behaviors with immunoreactivity (Rossi et al., 2011). This finding that the autoantibodies are also present in typically developing children suggests that in ASD, there may be some other pathological mechanism that is allowing the autoantibodies to enter the typically “immune-privileged” brain, contributing to ASD behaviors (Rossi et al., 2011). Using human protein extracts as antigenic targets the same researchers found antibody reactivity to CNS proteins at two separate molecular weights (45 and 62 kDa) that correlated with worsening behaviors in children with ASD (Goines P. et al., 2011). Autoantibodies specific for a 45 kDa cerebellar protein were associated with a diagnosis of autism disorder (Goines P. et al., 2011) and cognitive impairment (Piras et al., 2014), while autoantibodies directed toward the 62 kDa protein were associated with the broader diagnosis of ASD (Goines P. et al., 2011) and motor stereotypies (Piras et al., 2014). A Saudi Arabian cohort of children with ASD also showed high levels of autoantibody reactivity to cerebellar neurons, the presence of which was positively associated with ASD severity (Mostafa and Al-Ayadhi, 2012b). Increased serum autoantibodies against human neuronal progenitor cell (NPC) proteins of 55, 105, 150 and 210 kDa molecular weights in ASD have also been identified, with the strongest reactivity noted in neuronal progenitor cells expressing the mature neuronal marker Tuj1, as opposed to astrocytes expressing Glial fibrillary acidic protein (GFAP) (Mazur-Kolecka et al., 2014). This group had previously found that sera from ASD subjects suppressed differentiation and maturation of NPCs in culture and provided a potential mechanism for aberrant neurodevelopment in ASD (Mazur-Kolecka et al., 2007, 2009, 2014).

Serum antibodies to ganglioside M1, the most abundant sialylated glycosphingolipid component of neuronal membranes, were found to be significantly higher in children with ASD compared to controls, with highest levels seen in the most severe cases of ASD (Mostafa and Al-Ayadhi, 2011). Serum autoantibodies to gangliosides are frequently seen in autoimmune disorders associated with neurological impairment, such as SLE and Guillain-Barré syndrome (Mostafa et al., 2010b; Kusunoki and Kaida, 2011). Additional autoantibodies identified in individuals with ASD include those reactive to cardiolipin, phosphoserine, and β2-glycoprotein 1 (Careaga et al., 2013), endothelial cells (Zhang et al., 2010; Bashir and Al-Ayadhi, 2015), myelin-associated glycoprotein (Mostafa and Al-Ayadhi, 2012a, 2013), double stranded DNA, nucleus and nucleosomes (Al-Ayadhi and Mostafa, 2014; Mostafa et al., 2014) and mitochondrial DNA (Zhang et al., 2010). Recently, folate receptor autoantibodies (FRA) have come to the forefront of autoantibody studies in children with ASD. In 2013, Frye et al. found FRAs to be prevalent in children with ASD (75%), including blocking and binding FRAs, with 29% being positive for both FRAs (Frye et al., 2013). This study additionally looked at supplementation of folinic acid, as FRA may be interfering with folate transport across the blood-brain barrier, and found improvement in communication, language, attention and stereotypic behaviors in treated children compared with non-supplemented ASD controls (Frye et al., 2013). In support of these findings, a Belgium study found significantly higher prevalence of blocking FRA in ASD compared to non-autistic individuals with developmental delays (Ramaekers et al., 2013). Both studies also found a statistically significant increase in FRAs among the parents of individuals with ASD, suggesting a relationship with familial autoimmunity. However, not all parents harbored these autoantibodies, and this suggests that in some instances there is postnatal acquisition of FRA (Frye et al., 2013; Ramaekers et al., 2013). 3-Chlorotyrosine, a marker of myeloperoxidase protein damage and inflammation, was significantly lower in those positive for blocking FRAs, suggesting that this group may have less inflammation than their counterparts positive for binding FRAs. Moreover, the presence of blocking FRAs in children with ASD was associated with less severe ASD symptoms compared to those who were negative for blocking FRA (Frye et al., 2016). Further studies characterizing immune activation in the different FRA groups could help clarify this relationship.

It is important to note that although the presence of autoantibodies are commonly found in autoimmunity, they may not be specific to any single disorder and can be present to some degree in healthy individuals, therefore they are not diagnostic without direct or indirect evidence (Rose and Bona, 1993; Lacroix-Desmazes et al., 1998). It is currently unknown whether these autoantibodies found in individuals with ASD play a causal role in the etiology of the disorder. The lack of consistency in target antigens and wide heterogeneity of type and presence of these autoantibodies suggest they may in fact be epiphenomenon in at least some cases of ASD due to general immune dysregulation (Wills et al., 2009). Collateral damage can occur from unregulated or excessive inflammatory responses, causing subsequent epitope spreading which leads to the development of autoantibodies characteristically seen in autoimmunity (Vanderlugt and Miller, 1996).

### Aberrant innate immune responses

#### Neuroinflammation

One of the major advancements in ASD research in the last 10 years is evidence that active neuroinflammation is a significant component of ASD, including chronically activated microglia (Figure 2). Findings of increased microglial and astroglial activation in the cerebellum and various regions of the cortices, specifically increased HLA-DR and GFAP via immunostaining (Vargas et al., 2005) has prompted additional research in this area. These early findings included increases in proinflammatory cytokines in the CSF and within several regions of the brain such as macrophage chemoattractant protein (MCP)−1, a cytokine important for monocyte recruitment. Findings also included marked increases in CD68+ perivascular macrophages and monocytes, suggesting the possibility of monocyte infiltration, which is one of the markers considered when autoimmunity is suspected in MS and EAE models (van Der Valk and De Groot, 2000; Vogel et al., 2013). Additionally, a significant loss of Purkinje neurons in the cerebellum was noted in ASD subjects compared with controls, and anti-inflammatory cytokines were associated with degenerative Purkinje cells and cerebellar astroglia, suggesting an attempt to modulate inflammation in the presence of damaged tissue (Vargas et al., 2005).

Microglia were later characterized in post-mortem brain samples of ASD subjects, revealing alterations indicative of an activated microglia phenotype including increased somal volume, increased density, and amoeboid presentation in 9 of 13 ASD cases (Morgan et al., 2010). These alterations were not correlated with age and researchers found no co-localization of Interleukin 1 receptor, type I (IL-1R1) with the monocyte/microglia marker ionized calcium binding adapter molecule 1 (Iba-1). IL-1R1 is upregulated rapidly during acute inflammation, therefore this lack of increased co-localization suggests this is not an acute inflammatory event, rather a long-standing alteration in the brains of ASD subjects (Morgan et al., 2010). This group later found increases in spatial clustering of microglia to neurons in these brain samples, suggesting neuron-directed recruitment of microglia in ASD subjects (Morgan et al., 2012). Considered the resident innate immune cells of the brain, microglia colonize the brain during the early embryonic period and are essential to neurodevelopment, including involvement in angiogenesis (Fantin et al., 2010; Rymo et al., 2011), regulation of astrocytic differentiation from neuronal precursor cells (Nakanishi et al., 2007), synaptic pruning (Paolicelli et al., 2011) and clearance of newborn neuronal precursors destined for apoptosis (Sierra et al., 2010). When activated to an inflammatory phenotype, microglia secrete inflammatory cytokines including tumor necrosis factor-alpha (TNF-α), IL-1β and IL-6 and produce nitric oxide synthase (iNOS) (Reviewed in Smith et al., 2012). Although some microglia activation is required for productive neurodevelopment (Cunningham et al., 2013), chronic activation is associated with disease states (Smith et al., 2012). Furthermore, excessive activation can lead to cell death and abnormal or reduced connectivity (Rodriguez and Kern, 2011).

To gain a more specific picture of the pro-inflammatory cytokine milieu in the brains of ASD subjects, Li et al. (2009) further investigated cytokines associated with inflammatory responses in post-mortem tissue and found significantly increased pro-inflammatory cytokines including interferon gamma (IFNγ) associated with NK cells and T helper (T_H_)-1 activation (Li et al., 2009). In support of altered immune regulation and function in the brains of ASD subjects, recent transcriptome analyses of the superior temporal gyrus and cerebral cortex of postmortem samples indicated upregulation of genes involved in immunity and inflammation, including markers of activated microglia and pathways of innate immunity (Garbett et al., 2008; Voineagu et al., 2011) More recent transcriptome analyses of multiple cortical areas of ASD brains support and add to these findings. Large scale RNA sequencing revealed that the dysregulated co-expression module found in the brains of ASD subjects by Voigneau et al. was enriched for activation specific to the microglia, and showed increased expression of “immune-response” genes (Gupta et al., 2014). Correlating well with these findings, methylation studies have identified that immune-response genes in frontal cortex of individuals with ASD have hypomethylated CpG sites, causing increased transcription of inflammatory genes such as TNF-α, integrin and complement genes, and genes that encode transcription factors involved in microglial development (Nardone et al., 2014).

To allow for *in-vivo* study of individuals with ASD, Suzuki and colleagues utilized positron emission tomography (PET) analysis to assess binding values of the [^11^C](*R*)-PK11195 radiotracer that binds selectively to the mitochondrial 18 kDa translocator protein (TSPO), specifically targeting activated microglia. They found significantly increased binding values in several regions of the brain compared to controls, suggesting increased microglia activation in all regions analyzed compared to controls, including the cerebellum, several regions of the cortex, and the corpus callosum (Suzuki et al., 2013). These *in-vivo* findings support studies of increased microglia activation in post-mortem tissue; however, it is important to note that (1) the sample sizes were small and only included individuals with high-functioning ASD, (2) significant non-specific binding can occur with the [^11^C](*R*)-PK11195 radiotracer, and (3) researchers were unable to normalize binding values due to lack of a microglia-free reference region (Suzuki et al., 2013). These findings warrant additional *in-vivo* studies with larger sample sizes/additional ASD phenotypes, and ideally a more-specifically binding radiotracer.

#### Peripheral innate immune dysfunction

Aberrant innate immune responses are not restricted to the brain and CNS in individuals with ASD, alterations in circulating monocytes, dendritic cells and NK cells have also been identified (Figure 2). Early studies found an increased number of monocytes in the peripheral blood of children with ASD (Denney et al., 1996; Sweeten et al., 2003b), and elevated production of IFNγ, IL-1RA, and a trend for elevated IL-6 and TNF-α in whole blood cultures, suggesting increased activation of monocytes in individuals with ASD (Croonenberghs et al., 2002a). In support of these findings, a recent study found increased CD95, a marker of activation on monocytes in the peripheral blood of children with ASD (Ashwood et al., 2011a). Sweeten and colleagues also found elevated plasma neopterin, a pyrazinopyrimidine compound produced by monocytes and macrophages in response to IFNγ stimulation, indicating increased cellular immune activation (Murr et al., 2002; Sweeten et al., 2003b). Additionally, after TLR2 and TLR4 stimulation, upregulation of inflammatory cytokines and the HLA-DR activation marker was seen in monocytes from children with ASD versus typically developing children (Enstrom et al., 2010). Dendritic cell numbers were also increased in children with ASD and associated with bigger amygdala size and more aberrant behaviors (Breece et al., 2013).

An early study investigating induced responses of immune cells in children with ASD found increased production of the innate cytokines: TNF-α, IL-1β, and IL-6 after stimulation of peripheral blood mononuclear cells (PBMC) with the TLR-4 ligand lipopolysaccharide (LPS), from children with ASD when compared to typically developing children (Jyonouchi et al., 2001). To improve understanding of differential innate responses to varied TLR stimuli in ASD, investigators measured innate responses to several environmentally relevant pathogen-associated molecular patterns (PAMPs). The outcome of this study demonstrated elevated cytokine production after exposure to several innate immune ligands. Stimulation of isolated monocytes with TLR2 ligand lipoteichoic acid (LTA) produced a significant increase in production of TNF-α, IL-1β, and IL-6 in children with ASD versus typically developing controls, supporting earlier works. TLR4 stimulation with LPS also produced increased IL-1β. Moreover, increased production of IL-1β after LPS stimulation was found to be associated with worsening behaviors (Enstrom et al., 2010). Recently, Nadeem et al. identified increases in the IL-17RA receptor on circulating monocytes in children with ASD (Nadeem et al., 2018). IL-17RA is the receptor for IL-17A, a cytokine associated with autoimmunity and implicated in rodent models of ASD (Choi et al., 2016). Increased expression of the nuclear transcription factor NFkB and inducible nitric oxide synthase (iNOS) were also noted in ASD groups. Cells treated with IL-17 increased expression of iNOS/NFkB and blockade of IL-17 reversed this inflammatory profile (Nadeem et al., 2018). These data may suggest a link between adaptive arm of the immune system and innate immune dysfunction in people with ASD.

Significant increases in cytokines associated with innate inflammation have also been found in phytohemagglutinin (PHA) stimulated PBMC of children with ASD, including TNF-α and GM-CSF (Ashwood et al., 2011b). Excessive production of pro-inflammatory cytokines initiated by the innate immune system could have downstream consequences including over-activation of the adaptive arm, leading to autoimmune sequelae. Abnormal innate cytokines have also been identified in the plasma and sera of individuals with ASD. Significant increases in plasma levels of IL-1β, IL-6, and TNF-α suggest increased activation of the innate arm (Emanuele et al., 2010; Ashwood et al., 2011b; Suzuki et al., 2011; Ricci et al., 2013) and are consistent with the dynamic responses seen previously in stimulated monocytes (Enstrom et al., 2010; Ashwood et al., 2011c). Other innate-associated cytokines reported to be elevated in the plasma or sera of individuals with ASD when compared to typically developing controls include IL-12p40 and the chemokines IL-8, MCP-1, regulated on activation, normal T cell expressed and secreted (RANTES), eotaxin and C-X-C motif chemokine 5 (CXCL5) (Ashwood et al., 2011b; Suzuki et al., 2011; Mostafa and Al-Ayadhi, 2015). While there have been a few contradictory reports regarded plasma/sera cytokine concentrations individuals with ASD, a recent meta-analysis of plasma cytokines showed significant evidence of abnormal cytokine/chemokine profiles in individuals with ASD versus healthy controls, including elevated IL-1β, IL-6, IL-8, IFN-γ, and MCP-1, and reduced concentrations of the anti-inflammatory cytokine transforming growth factor beta 1 (TGFβ1) (Masi et al., 2015). Further characterization of ASD subjects found that circulating plasma levels of pro-inflammatory cytokines associated with increased innate immune activation correlated with worsening behaviors, which suggests that ongoing inflammation likely contributes to the severity of behaviors (Ashwood et al., 2011b). Leukocyte adhesion molecules have also been investigated in children with ASD, and were found to be reduced, indicating dysfunctional immune-endothelial cell interactions that could have implications for the migration of innate immune cells into the CNS (Onore et al., 2012).

NK cells are important early responders of the innate immune system. They specifically target virally infected cells and play important roles in both tumor surveillance and protection of the fetus during pregnancy. As early responders, they can initiate a cascade of immune responses and if dysfunctional these important signals may be missing or altered (Mandal and Viswanathan, 2015). A significant increase of total numbers of NK cells, identified as CD56^+^CD3^−^, was observed in children with autism with both high and low IQ (Ashwood et al., 2011a). Cytokines produced by NK cells expressing high levels of CD56 can significantly influence the cytokine milieu. Upregulation of mRNA responsible for expression of receptors including killer-cell immunoglobulin-like receptors [KIRs] and increased cytokine, perforin, and granzyme B production was observed at resting levels in NK cells from 2 to 5 year old children with ASD (Enstrom A. M. et al., 2009). The cytolytic function of NK cells is important for immune regulation, as they can remove persistently activated immune cells (Cook et al., 2014). Interestingly, when stimulated, significantly decreased cytotoxicity and lower production of effector molecules (granzyme, perforin, and IFNγ) were seen in children with ASD compared to controls (Enstrom A. M. et al., 2009). Decreased cytolytic activity was seen previously in a large subgroup of children with ASD (Vojdani et al., 2008). This pattern suggested that NK cell activation may be “maxed-out” *in vivo*, and the cells may be unable to respond to further stimuli. Similar patterns of increased CD56^+^ NK cells but impaired cytolytic activity have been seen in the peripheral blood of patients with autoimmune disorders such as MS, T1DM, SLE, and RA (Fogel et al., 2013), again adding evidence of an autoimmune component/lack of immune regulation in individuals with ASD.

### Aberrant adaptive immune responses

#### T cells

Over the last 10 years, researchers have found significant abnormalities of the adaptive arm of the immune system in people with ASD, including altered numbers of lymphocytes, dysregulation of T and B cell activation, and altered adaptive cytokine production (Figure 2). Increased total numbers of T cells and skewed ratios of CD4 to CD8 lymphocytes have been associated with decreased executive function in people with ASD (Han et al., 2011). Several studies have also shown altered cytokine production in T cells. Molloy et al. found a shift to a T_H_2 phenotype with significant increases in IL-4, IL-5, and IL-13 after stimulation *in vitro* (Molloy et al., 2006b). Altered surface markers of T cell activation have also been seen. Specifically, the T cell activation markers HLA-DR and CD26 were found to be increased in children with ASD (Ashwood et al., 2011a). CD5, a transmembrane protein associated with T cells and found to be elevated in autoimmunity (Sigal, 2012), was recently found to be significantly elevated in plasma of ASD subjects, and is associated with worsening severity of ASD (Halepoto et al., 2017). In response to stimulation, T cells from children with ASD also showed increased CD134 and increased cellular proliferation associated with worsening behaviors (Ashwood et al., 2011b). CD134 (also known as OX40) is a co-stimulatory molecule expressed on activated T cells, including memory T cell subsets (Webb et al., 2016) that is required for optimal activation of naïve T cells and is important in survival and maintenance of memory T cells.

Significant alterations in cytokines associated with the adaptive arm have been found in children with ASD versus controls, including IL-5, IL-13, IL-17 (Suzuki et al., 2011), IL-23 and IL-12 (Ricci et al., 2013), IL-21 and IL-22 (Ahmad et al., 2017a). Two recent studies found altered cytokine profiles in neonatal blood spots, suggesting early immune dysregulation. Increased IL-4 at birth was associated with increasing severity of ASD, and increased IL-1β with milder versions of ASD (Krakowiak et al., 2017). Zerbo et al. observed increased MCP-1 and decreased RANTES at birth in children with ASD (Zerbo et al., 2014). It is noteworthy to mention that various reports of T cell skewing in ASD does not necessarily implicate a specific polarization associated with the disorder, rather it supports the suggestion that a lack of regulation may be at play. A recent study clustered subjects into immune endophenotypes based on T cell polarization after stimulation with PHA, and found that both T_H_1 and T_H_2 responses were associated with worsening behaviors and increased severity of core ASD symptoms (Careaga et al., 2017). Transcription factors associated with inflammatory T cell activation, and different T cell subsets, namely T-box transcription factor (Tbet), GATA binding protein 3 (GATA3) and retinoid-acid receptor-relat-ed orphan receptor gamma t (RORyT) are all increased in children with ASD (Ahmad et al., 2017b). These studies support the notion of distinct clusters of ASD phenotypes characterized by immune dysfunction (Sacco et al., 2010, 2012).

Aberrant T cell responses or decreased removal of activated T cells can lead to autoimmune pathology (Joller et al., 2012). CD95 is the first apoptosis signal (Fas) receptor, which initiates apoptosis of activated T cells when they are repeatedly exposed to antigen. It has critical importance in tolerance and regulation, and alterations in Fas signaling may play a role in the development of autoimmunity (Siegel and Fleisher, 1999). Reduced CD95 expression on T cells from ASD subjects compared to controls is suggestive of decreased apoptosis of potentially overactive T cells in ASD (Engstrom et al., 2003).

#### Regulatory T cells

One of the most important immune components in the prevention of autoimmunity is regulation, and regulatory T cells (T_regs_) play a key role in immune regulation and homeostasis (Sakaguchi, 2004). Humans that carry mutations in the transcription factor forkhead box P3 (FOXP3) or have depletion in T_regs_ develop severe autoimmunity (Sakaguchi et al., 1995; Miyara et al., 2005; Toubi et al., 2005; Long and Buckner, 2011; Fujio et al., 2012). T_regs_ have been found to be critical for preventing autoimmunity in murine models (Sakaguchi et al., 1995) and a deficiency of T_regs_ may play a role in the development of autoimmune disorders, including RA (Toubi et al., 2005) and SLE (Miyara et al., 2005). Autoimmune pathology can occur when immune regulation breaks down, disrupting tolerance and homeostasis, and leading to an aberrant attack on self (Lourenço and La Cava, 2011). Typically in autoimmunity, a breakdown in tolerance will lead to the production of destructive autoantibodies by plasma cells and self-reactive T cells with a deficit in number or activity of T_regs_ (Sakaguchi, 2004). Notably, several studies have found that T_regs_ or their regulatory effector molecules are decreased in some individuals with ASD (Figure 2) (Okada et al., 2007; Ashwood et al., 2008; Mostafa et al., 2010a). For example, plasma TGFβ1 was significantly reduced in adult males with Asperger's syndrome (Okada et al., 2007) and in children with ASD (Ashwood et al., 2008). Reduced TGFβ1 was associated with increased ASD severity and lower adaptive and cognitive behaviors (Ashwood et al., 2008). Furthermore, microRNAs (miRNAs) involved in controlling TGFβ1 signaling pathways show differential expression in the cortex and serum of individuals with ASD (Mundalil Vasu et al., 2014; Ander et al., 2015; Huang et al., 2015). Additionally, circulating CD4^+^CD25^high^ T_regs_ were found to be significantly decreased in children with ASD, with reduced frequency correlating with severity of the disorder (Mostafa et al., 2010a). Several studies have also shown decreased IL-10 production after stimulation of CD4+ T cells (Jyonouchi et al., 2001, 2005, 2014; Ashwood and Wakefield, 2006).

#### B cells

Antibody production of high specificity to various antigens is the primary role of B cells, in order to neutralize and help eliminate pathogens. Despite the growing number of studies identifying autoantibodies in subjects with ASD, the B cells responsible for antibody production have been poorly studied. Irregularities in B cell populations and antibody production have been identified in a small number of ASD studies, although with conflicting results. A 2011 study found increased numbers of total (CD20^+^) and activated (CD38^+^) B cells in children with ASD compared to age-matched controls. No differences were seen in naïve (CD5^+^) B cells, thus the increase in total cells was likely due to increased activated cells, suggesting increased immune activation overall (Ashwood et al., 2011a). Higher numbers of CD19/CD23 B lymphocytes were also found in children age 3–6 recently diagnosed with regressive autism, supporting the previous findings (Wasilewska et al., 2012). However, Heuer et al. found no differences in total numbers of B cells, and B cell responses to stimulation were not different among ASD subjects compared to controls (Heuer et al., 2012). Differences in study design and markers used (CD20 and CD19, respectively) could account for the contradictory results. Neither marker is comprehensive for individual B cell subsets as their expression is decreased as B cells mature and differentiate into antibody-secreting plasma cells (Tedder, 2009). CD38 expression increases significantly upon maturation, therefore this marker may be a better indicator of the population of effector cells that may be responsible the production of specific autoantibodies. Additional research is needed to further characterize these cells, including identifying populations of positive regulators and B-regulatory cells (B_regs_), as these cells secrete IL-10, have recently been found to play a role in the induction of T_regs_ (Fujio et al., 2013), and play an important role in the acquisition of tolerance during pregnancy (Rolle et al., 2013).

In addition to significant levels of autoantibodies found in individuals with ASD, atypical antibody production has been frequently seen in ASD serum and plasma with correlations to behaviors. Results vary and are often contradictory—these inconsistencies may be due to small sample sizes and improper controls such as “population standard” versus age-matched controls residing in the same locale, and lack of adjusting for seasonality. Comparing immunoglobulin levels across a broad age range can produce inconsistencies, thus it is critical to have age-matched controls. For example, an early report found decreased circulating IgA associated with HLA-DR antigens in a subset of ASD subjects (Warren et al., 1997), and a 2012 study supported these findings (Wasilewska et al., 2012); however, other studies showed no change in IgA (Heuer et al., 2008). Ages varied widely in the Warren study (from ages 5 to 31) and may account for discrepancy because IgA does not reach adult levels until around age 10 (Aksu et al., 2006) whereas the later studies were age-matched. Decreased IgA in children with regressive ASD did not fulfill the criteria for either partial or full IgA deficiency in the study by Wasilewska and colleagues, and likely reflects immune dysfunction in a subset of patients (Wasilewska et al., 2012). Heuer et al. found that decreased plasma IgG/IgM negatively correlated with worsening behaviors as assessed using the Autism Behaviors Checklist (ABC) (Heuer et al., 2008). Researchers also found a significant increase in IgG4 in ASD subjects, with a trending increase in IgG2 subtype (Croonenberghs et al., 2002b; Enstrom A. et al., 2009). IgG4 is a blocking antibody, produced under conditions of chronic antigen exposure and class switch to IgG4 is dependent on T_H_2 cytokines (IL-4/IL-13) (Aalberse et al., 2009). This is consistent with the dynamic T cell responses found by later by Ashwood et al. (2011c). These correlations support an association with immune dysfunction and potentially a lack of immune regulation in individuals with ASD.

## Improvement of symptoms with the use of immune-modulating drugs and supplements

Several clinical trials have shown the efficacy of immunosuppressive drugs for improving behaviors in individuals with ASD. The first study to show this relationship used corticosteroid treatment in a 6-year-old boy with language regression at 22 months who received a diagnosis of PDD (pervasive developmental disorder) at age 3-1/2. After several weeks of this treatment, the boy experienced significant gains in expressive language and responsiveness to communication to nearly age-appropriate levels, and reduction in stereotypical echolalia (Stefanatos et al., 1995). Since then, improvements have been seen in language ability, behaviors and motor development in several case studies and clinical trials using corticosteroids and immunosuppressive drugs (Mott et al., 1996; Chez et al., 1998; Shenoy et al., 2000; Mordekar et al., 2009) including a recent study that showed improvement in language-specific electrophysiological brain function after treatment with corticosteroids (Duffy et al., 2014). The effectiveness of the anti-psychotic medication risperidone in improving stereotyped behaviors and social withdrawal in individuals with ASD was increased with the addition of celecoxib, a cyclooxygenase-2 (COX-2) selective nonsteroidal anti-inflammatory drug (Asadabadi et al., 2013). Human cord blood mononuclear cell transplantation, alone and in combination with umbilical mesenchymal stem cell transplantation significantly improved behaviors in children with ASD compared to controls. These transplanted cell types are known to have profound immune-regulatory capabilities, suggesting that a possible mechanism of improvements seen may be immune-modulation (Lv et al., 2013). These trials and cases studies, although small and specific to select groups of ASD subjects, suggest that in some children with ASD, immune abnormalities may be driving certain behaviors.

Nutritional or supplemental approaches may be helpful in modulating immune function in people with ASD. Several studies have found children with ASD to be deficient in serum levels of vitamin D (25-hydroxycholecalciferol), and serum levels were found to be negatively associated with language and behavioral scores on the ABC and the Childhood Autism Rating Scale (CARS) (Desoky et al., 2017; Saad et al., 2018), (Feng et al., 2017). Serum vitamin D levels also negatively correlated with the presence of anti-myelin-associated glycoprotein autoantibodies (Mostafa and Al-Ayadhi, 2012a). This fat-soluble vitamin has important immunomodulatory and neuroprotective functions (Aranow, 2011; Wrzosek et al., 2013), and deficiency may be contributing to immune and behavioral abnormalities in people with ASD. Daily supplementation with Vitamin D, not to exceed 5,000 IU/day, was found to significantly improve behavioral outcomes and lowered elevation of CD5 expression in children with ASD, supporting a role for Vitamin D in modulating the immune system (Desoky et al., 2017; Feng et al., 2017; Saad et al., 2018). As previously discussed, children with ASD have altered T cell profiles, perhaps due to altered transcriptional activity (Ahmad et al., 2017b). Similar T cell alterations have been seen in the inbred BTBR mouse model of ASD, and a recent study showed modulation of this transcriptional activity through the administration of the antioxidant resveratrol, a nutritional component found in various fruits, legumes, and grape juice. Resveratrol increased mRNA expression of Foxp3 in spleen and brain tissues, and increased the number of Foxp3^+^ T regulatory cells in the periphery of BTBR and B6 mice. Resveratrol also decreased expression of transcription factors associated with inflammatory T cells, including T_H_17 cells. Additionally, the nutritional compound decreased ASD associated repetitive behaviors in BTBR mice (Bakheet et al., 2017). These studies, offer support that immune dysfunction is driving at least some of the pathological outcomes in subsets of people with ASD. As our understanding of how immune dysregulation is contributing to the pathogenesis of ASD grows, more treatments can be targeted specifically to these mechanisms.

## Outstanding questions and conclusion

**Figure 3:**
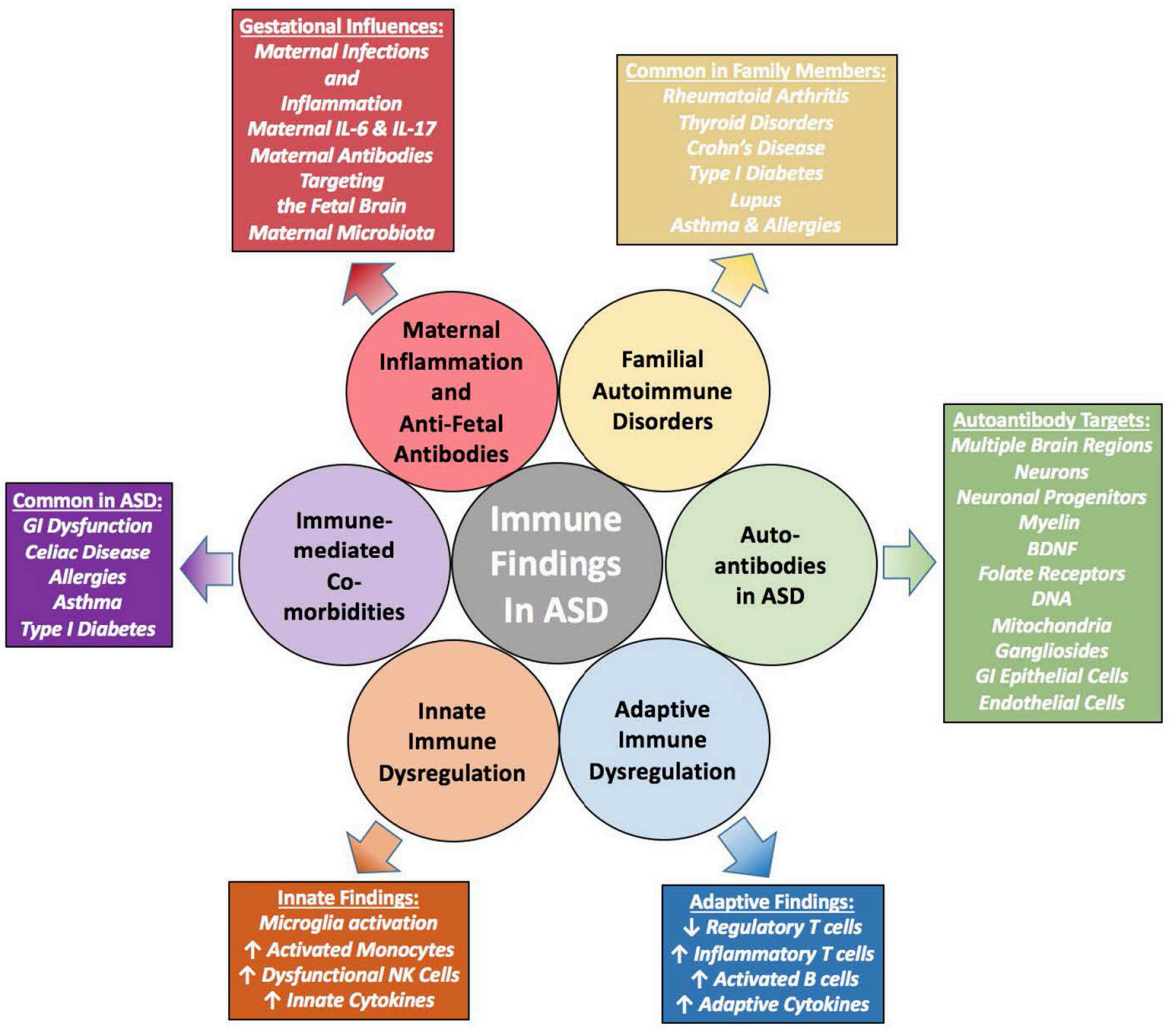
Summary of Immune Evidence in ASD–is Immune Dysregulation Causing or Contributing to these Disorders? Immune findings in individuals with ASD have grown from a few scant early studies to a plethora of extensive and varied research showing immune dysfunction that contributes to worsening behaviors. Familial autoimmunity is a common finding within families affected by ASD. In addition, individuals with ASD have significant immune dysregulation that contribute to altered behaviors. These individuals also suffer more so than the general population from immune-mediated comorbidities such as allergies, asthma and gastrointestinal (GI) disturbances. Mechanistically, studies have shown that the gestational immune environment must be delicately balanced, and without such balance neurodevelopment can be altered. Whether these immune characteristics are causal or just sequelae of the overarching disorders remain to be determined; however, the evidence is building that the dysregulated immune response may be pathologically contributing to ASD.

The evidence that immune dysfunction likely plays a role in the etiology/pathophysiology of ASD is becoming substantial. Familial autoimmunity is a common risk factor, and maternal autoantibodies and inflammation during gestation significantly increase the risk of having a child with ASD. Furthermore, individuals with ASD have significant immune dysfunction and inflammation. They also suffer from immune-mediated co-morbidities much more often than the typically developing population, including GI dysfunction and dysbiosis. The presence of autoantibodies in individuals with ASD is increased, and evidence of neuroinflammation has been substantiated both *in vivo* and in post-mortem brain tissue. Although the plethora of evidence identifying a connection between autoimmunity, immune dysfunction, and ASD (summarized in Figure 3) is tantalizing, it still leaves many mechanistic questions regarding the impact of immune system dysfunction on the development of ASD.

The most outstanding question remaining from these studies is whether the immune dysfunction is causal or rather sequelae of the larger disorder. The origin of the immune dysfunction seen in many individuals with ASD and the role it plays in the aberrant behaviors is still unknown, although many of these studies discussed throughout this review support an association of worsening behaviors associated with altered immune function. Gestational influences, including maternal immune activation and the presence of maternal autoantibodies may be contributing to altered early neurodevelopment and immune dysfunction in offspring, and these are supported by preclinical animal models of both maternal immune activation and passive transfer of autoantibodies. Interactions between the different immune cells leading to inflammation and altered cytokine production in people with ASD may be directly contributing to abnormal brain development and signaling, and the ever expanding knowledge of neuro-immune cross-talk may eventually elucidate some of the mechanisms involved in the pathogenesis of ASD.

To date, categorizing ASD and immune dysfunction has been a difficult task due to the heterogeneity of the disorder and the changing diagnostic criteria; however, the recent focus on clustering phenotypes may provide a clearer picture to help elucidate the different factors involved in the etiologies of these complex disorders. The immune dysfunction driving the development of autoantibodies and overall immune abnormalities in people with ASD remains unknown, however, recent insights into dysbiosis causing aberrant immune system education could be a plausible mechanism as to the origin of immune dysfunction. Prenatal immune influences could be driving direct and/or epigenetic changes in gene expression responsible for altered neurodevelopment. As future studies improve our understanding of these complex and interconnected systems, it will allow for development of new therapies that target immune dysfunction in ASD. Future research could focus on interventions that improve immune parameters to help identify mechanisms involved in development and exacerbation of ASD symptoms. As our understanding of the involvement of the immune system in ASD grows, it can shape future hypotheses and research to better identify the pathological mechanisms involved.

## Author contributions

HH wrote the first draft of the manuscript. EM, DR, and PA wrote sections of the manuscript. All authors contributed to manuscript revision, read and approved the submitted version.

### Conflict of interest statement

The authors declare that the research was conducted in the absence of any commercial or financial relationships that could be construed as a potential conflict of interest.
